# Simultaneous decolorization of anionic and cationic dyes by 3D metal-free easily separable visible light active photocatalyst

**DOI:** 10.1007/s11356-022-22838-8

**Published:** 2022-09-09

**Authors:** Eman S. Mansor, Fatma N. El Shall, Emad K. Radwan

**Affiliations:** 1 Water Pollution Research Department, National Research Centre, 33 El Buhouth St, Dokki, Giza, 12622 Egypt; 2 Dyeing, Printing and Textile Auxiliary Department, Textile Research and Technology Institute, National Research Centre, El-Buhouth St. 33, Dokki, Cairo, 12622 Egypt

**Keywords:** Methylene blue, Methyl orange, Graphitic carbon nitride, Polyurethane foam, Wastewater treatment, Heterogeneous photocatalysis

## Abstract

To overcome the hard and costly post-treatment separation of ultrathin graphitic carbon nitride nanosheets (UGCN), it was supported on polyurethane foam (PUF). The ratio of PUF/UGCN was optimized for the removal of a mixture of methylene blue (MB) and methyl orange (MO) dyes. The characteristics of the composite photocatalyst and its photocatalytic performance were detailly studied. The X-ray diffraction and Fourier transform infrared results proved the successful preparation of UGCN and PUF and that the PUF/UGCN composite combines the features of both pure materials. The transmission electron microscopy illustrated the ultrathin nanosheet shape of the UGCN, while the scanning electron microscope showed the highly porous 3D-hierarchical structure of PUF. Compared to the pure components, the composite photocatalyst with PUF/UGCN mass ratio of 4 achieved better decolorization of MO and almost same decolorization of MB as UGCN. Neutral pH and 1 g/L of the composite photocatalyst were the optimum conditions for MB/MO mixture decolorization. The composite photocatalyst kept its efficiency for five successive cycles. Hydroxyl radicals were the dominant in the degradation of MB, while superoxide radicals were the most influencer in MO degradation. Conclusively, supporting UGCN onto PUF kept the photocatalytic efficiency of UGCN toward MB decolorization and improved its efficiency toward MO. Moreover, it enabled the reuse of the composite photocatalyst and facilitated the post-treatment separation process.

## Introduction

Water resources pollution is currently a global environmental challenge. The main source of water resources pollution is the discharge of untreated or partially treated industrial, agricultural, and/or domestic wastewater (Radwan et al. 2021; Radwan et al. 2020). These wastewaters contain a variety of organic and inorganic contaminants (Liu et al. 2021). Synthetic organic dyes are one of the largest groups of organic contaminants because of their extensive use in several industries (Kasumov et al. 2021; Nour et al. 2021). The features of synthetic dyes such as bio-recalcitrance, toxicity, carcinogenicity, mutagenicity, and light absorbance make them harmful to humans and aquatic environment (Kasumov et al. 2021; Nour et al. 2021; Zhang et al. 2021). Therefore, several treatment technologies for the removal of synthetic organic dyes from contaminated water have been evaluated and developed (El Bendary et al. 2021). Among these treatment technologies, heterogenous photocatalysis is a promising one because of its high efficiency, ecofriendly, and safety (Liu et al. 2019).

Graphite carbon nitride (GCN) is a metal-free organic polymeric nanomaterial that can be prepared from low-cost materials such as melamine and urea by thermal polymerization. It has proven outstanding performance in several fields including photocatalytic degradation of water contaminants owing to its unique features such as moderate band gap, thermodynamic suitability, physicochemical stability, high polymeric flexibility, super optical characteristic, and photoelectric properties (Cheng et al. 2021; Ren et al. 2021; Wang et al. 2021). However, one of the drawbacks, similar to other nanomaterials, is the difficult and costly separation of GCN from water after the treatment process due to its nano-size. To overcome this drawback immobilization on a support is attractive strategy that can enable ease separation and reuse for long-term effectiveness and durability (Li et al. 2015; Pattanayak et al. 2021; Radwan et al. 2018; Wang et al. 2017).

In spite of the fact that immobilized photocatalysts suffer from lower surface area and usually have lower efficiency than the slurry counterparts (Ainali et al. 2021), supporting nano-sized photocatalysts on a low-density material to get a floating photocatalysts become attractive recently. Floating photocatalysts have several advantages such as facilitating the post-treatment separation process, improving the utilization of solar light (no loss due to transmission through aqueous phase), and the reaction at the aqueous/air interface provides sufficient O_2_ and H_2_O (Li et al. 2015).

Polyurethane foam (PUF) is a low-cost three-dimensional (3D) porous flexible organic polymer. It is produced on an industrial scale and has several applications such as supporting material (for example in freezers and refrigerators), building insulation, furniture, packaging and bedding, automotive applications, water treatment, catalysis and oil-water separation. The pore size, densities, morphologies, and rigidities of PUF are manageable. Therefore, a PUF with tailored properties for a specific application can be obtained by controlling the preparation conditions. A PUF with a low density that allows it to float on the surface of an aqueous reaction system and absorb more solar light, open skeleton, high surface area, and natural hydrophobicity is an excellent support to immobilize photocatalysts (Li et al. 2018; Li et al. 2015; Pattanayak et al. 2021; Wang et al. 2017).

Herein, we aimed at the preparation of a floating easily separable visible light active photocatalyst that can efficiently and simultaneously remove anionic and cationic dyes from contaminated water. Pure bulk GCN was prepared by thermal polymerization of melamine followed by exfoliation into ultrathin nanosheets. Afterward, PUF and UGCN supported onto PUF (PUF/UGCN) with different ratios were prepared by a catalytic two-step method. The prepared materials were characterized by X-ray diffraction (XRD), attenuated total reflection-Fourier transform infrared (ATR-FTIR), high-resolution scanning electron microscope (HR-SEM), UV–vis diffuse reflectance spectroscopy (DRS), and transmission electron microscopy (TEM). The photocatalytic activity of the prepared materials for the decolorization of a mixture of anionic and cationic dyes was evaluated. Methyl orange (MO) and methylene blue (MB) were selected as models for anionic and cationic dyes, respectively, because of their wide use in several industries such as pharmaceutical, textile, and food (Fagier 2021). The effects of contaminated water initial pH (pH_i_) and the amount of photocatalyst on the efficiency of the photocatalytic process were studied. In addition, the reusability of the photocatalyst and the mechanism of the photocatalytic reaction were investigated.

## Materials and methods

The trifunctional polyether polyol monomer Lupranol®2004/1 was supplied by Dow Chemical Company, USA. Dibutyltin dilaurate (DBTDL), methylene diphenyl diisocyanate (MDI) and N, N-dimethylformamide (DMF) were supplied from ACROS Chemical Co. Melamine (99%), methylene blue (99%), and methyl orange (99%) were provided by Sigma Aldrich. All chemicals were used as received.

### Preparation of UGCN

**Scheme 1:**
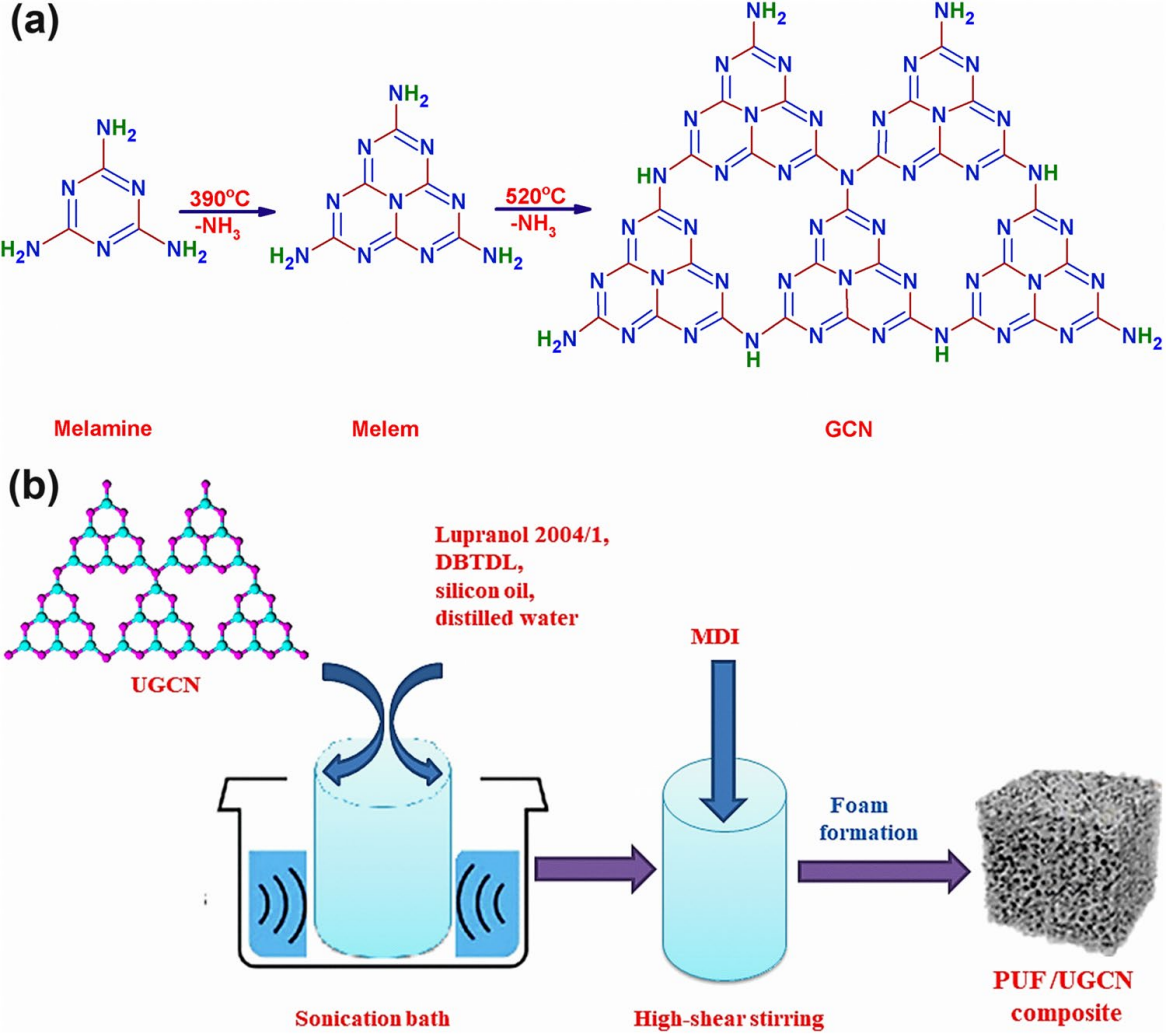
Preparation of **a** GCN by thermal polymerization of melamine and **b** PUF/UGCN composite

The GCN power was synthesized according to the literature (Yu et al. 2017b). Specifically, bulk GCN was prepared by heating 2 g of melamine powder for 2 h at 550 °C in a crucible. The thermal polymerization of melamine to bulk GCN is given in Scheme 1a. After cooling down to room temperature naturally, the resulting yellow product was collected and ground into powder for further use. The bulk GCN was exfoliated into ultrathin layers by dispersing in isopropanol (IPA) to form a 1 mg/mL suspension followed by sonication for 4 h. Ultrathin GCN nanosheets were obtained after centrifugation and freeze drying.

### Preparation of pure PUF

The preparation of pure and UGCN loaded PUF followed a two-component system procedure as depicted in Scheme 1b. The component A consisted of a mixture of calculated amounts of polyols, distilled water (DW, chemical blowing agent), dibutyl-tin dilaurate (DBTDL, catalyst), and silicon oil (surfactant). The mixture was subjected to a high-shear stirring at 3000 rpm for about 60–120 s. Component B was methylene diphenyl diisocyanate (MDI). The foaming reaction was carried out in one step by adding B to A (NCO to OH ratio = 2:1), followed by vigorous stirring for at least 15–90 s at room temperature; then, the system was poured directly into an open mold and was left without stirring to allow foam to rise up. After the foam was formed, it was left for about 6–12 h at room temperature to ensure its stability and complete hardening before further use.

### Preparation of PUF/UGCN composite

The component A was prepared by suspending different amounts of UGCN in DMF, followed by 5 min of sonication, and then, the UGCN suspension was mixed with polyether polyols at ambient temperature using a high shear stirrer. This suspension along with both DBTDL and silicon oil were subjected to sonication for 15 min. Subsequently, component B (MDI) was directly added to UGCN loaded component A and the procedure was continued as described in the preparation of pure PUF.

### Material characterization

X-ray diffraction (XRD) pattern in the 2*θ* range of 4–80° was recorded using PANalytical X’Pert PRO diffractometer (Cu Kα radiation, 40 mA and 45 kV). Attenuated total reflection-Fourier transform infrared (AT-FTIR) spectra of the prepared materials were recorded by JASCO FTIR 4100 spectrometer (Japan) in the range of 400–4000 cm^−1^ (with a resolution of 4 cm^−1^ and 60 scans). Scanning electron microscopy (SEM) micrographs of foam-based samples were captured by a Quanta FEG-250 microscope at a voltage of 20 kV. Prior analysis, the foam-based samples were sputter-coated with gold to avoid charging. Transmission electron microscopy micrographs of UGCN were captured using JEOL TEM-2100. A JASCO V570 spectrophotometer (Japan) was used to collect the UV–vis diffuse reflectance spectrum (DRS) of the prepared materials. The DRS were recorded in the wavelength range of 200–1000 nm, and the baseline was corrected using BaSO_4_ as the reference standard.

### Photocatalytic experiments

**Fig. 1:**
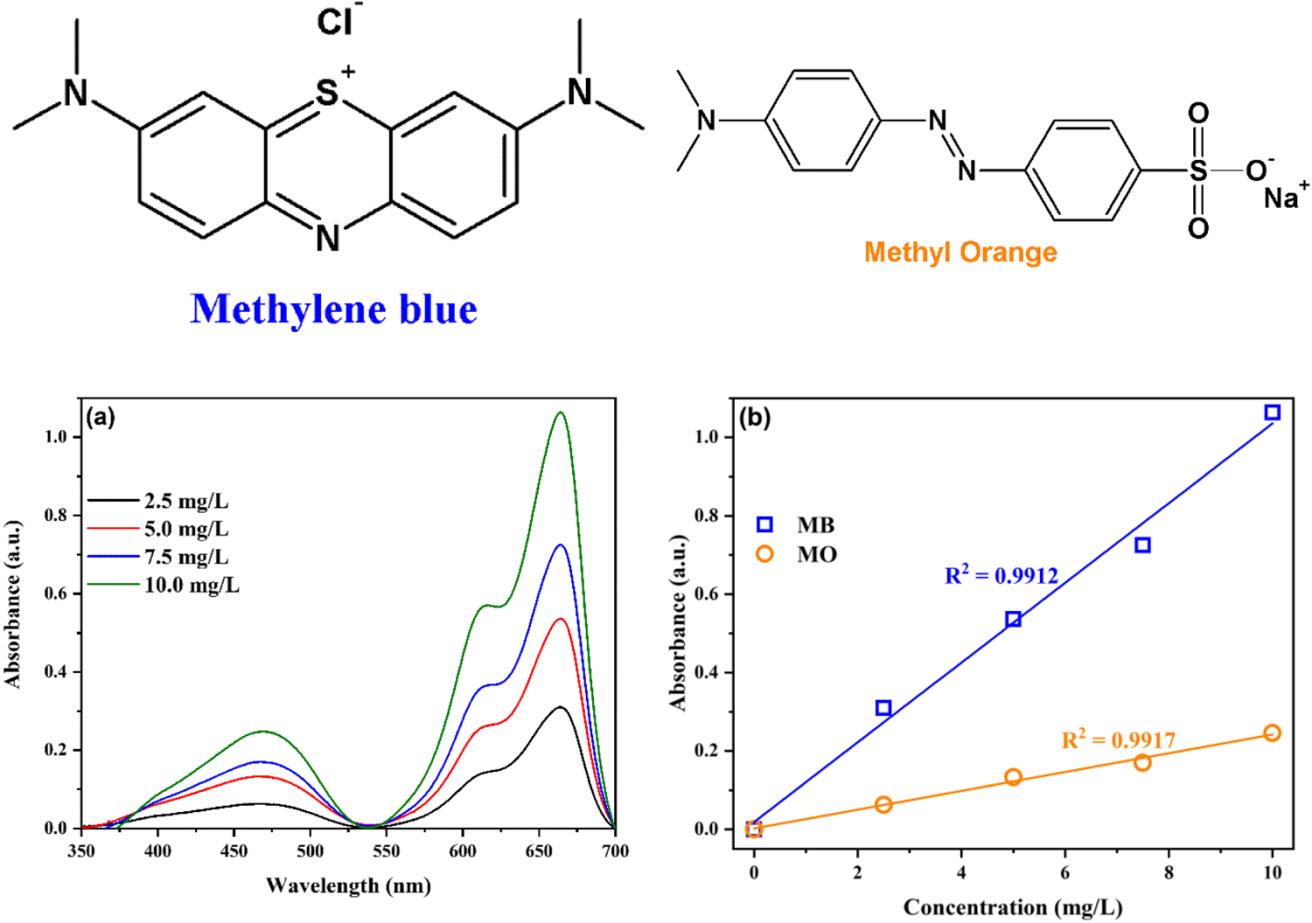
Chemical structures of MB and MO, UV-vis spectra of MB, and MO mixture at different initial concentrations (**a**) and their calibration curves (**b**)

In a typical experiment, 50 mg of the photocatalyst was dispersed into a beaker containing 50 mL of 7.5 mg/L of each of methylene blue (MB) and methyl orange (MO) mixed dye solution. After attaining the adsorption equilibrium via agitation in dark, the reaction was illuminated using a 200-W Xe lamp (200–2500 nm). Subsequently, 2.5 mL of dye solution was collected at specific time intervals and the concentrations of MB and MO were determined using Agilent Cary 100 UV/Vis spectrophotometer. Figure 1 shows the structures of the dyes, the UV-vis spectra of the dyes’ mixture at different initial concentrations (C_i_), and the corresponding calibration curves. The maximum wavelengths for the determination of MB and MO were 664 nm and 465 nm, respectively.

## Results and discussions

### Material characteristics

**Fig. 2:**
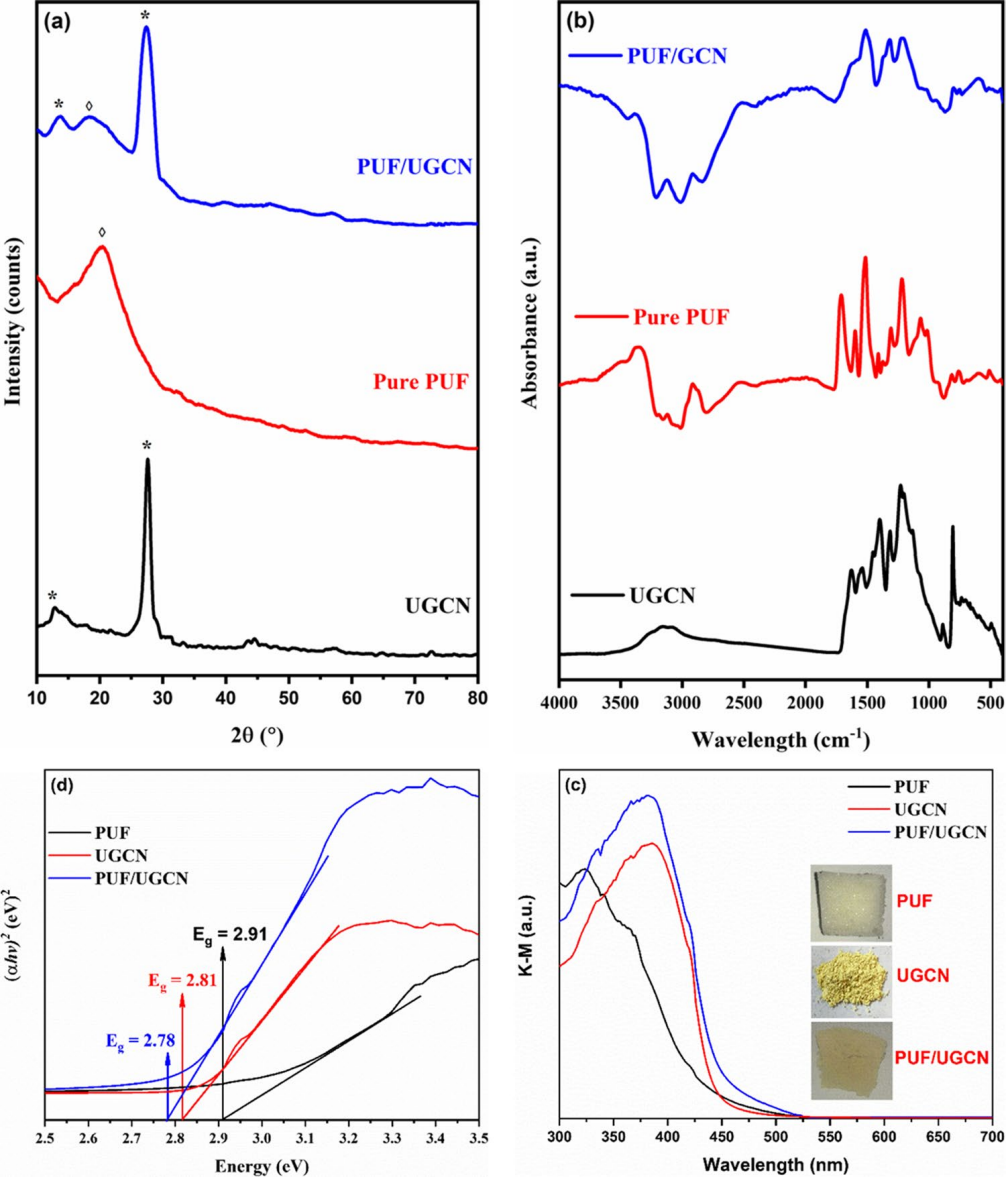
**a** XRD, **b** FTIR, **c** K–M conversion of the UV–vis DRS, and **d** Tauc plots of pure PUF, UGCN, and PUF/UGCN composite

The crystallinity of the pure and composite materials was evaluated by XRD; the obtained patterns are graphed in Fig. 2a. The XRD pattern of the UGCN prepared in this study is typical for UGCN reported in the literature and indicates the graphitic-like structure of the prepared UGCN. It has a main peak at 27.5° which corresponds to the interlayer stacking reflection (002) and a small peak at 13.0° which corresponds to the in-plane repeated units of tri-s-triazine (100) (Ma et al. 2017; She et al. 2017). On the other side, the XRD pattern of pure PUF shows the presence of a broad peak at 20.4° which indicates the amorphous nature of the prepared PUF. Notably, the obtained XRD pattern is typical for isocyanate-based PUF (Farukh and Dhawan 2016; Kim et al. 2018; Yuan et al. 2021). The composite material (PUF/UGCN) has three diffraction peaks at 13.5°, 19.0°, and 27.2°. The first and last peaks can be assigned to the UGCN, while the broad peak at around 19.0° can be assigned to the PUF. This result proves the coexistence of UGCN and PUF and indicates the successful preparation of the PUF/UGCN composite.

To get information about the surface functional groups of the prepared materials, FTIR was performed and the results are shown in Fig. 1b. In the FTIR spectrum of the as-prepared UGCN, the sharp peak at around 804 cm^−1^ is characteristic for the triazine units. The peaks in the range 890–1620 cm^−1^ are typical for the stretching vibration of C–N and C–NH heterocycles which include both repeating C−N(−C)−C (full condensation) and bridging C–NH–C units. The broad band at 3156 cm^−1^ arises from the stretching vibration of O–H of adsorbed moisture and N–H of uncondensed amino groups (Wang et al. 2020; Yang et al. 2017; Yu et al. 2017a). The FTIR spectra of PUF and PUF/UGCN showed that the characteristic functional group bands in both materials are almost the same, implying that the chemical structure of PUF foam has not affected by loading UGCN (Xu et al. 2007). The spectrum of pure PUF showed broad, strong peaks in the range of 3384–3121 cm^−1^ corresponding to N–H stretching of the urethane group. The absorbance spectra showed two vibrational bands in this range; these bands correspond to unbound and hydrogen bonded linkage. The hydrogen bonded band was located at a lower wavelength. The carbonyl (–C=O) of the urethane group and the ether (–C–O–C–) are the two functional groups that could act as acceptors with N–H in hydrogen bonding (Hejna et al. 2017; Xu et al. 2007). The bending vibrations of the N–H linkage appeared in the range of 1514–1506 cm^−1^. The band signals at 2914 cm^−1^ were for asymmetric and symmetric C–H bonds stretching vibrations (Hejna et al. 2017). The bands that appeared in the wavelength range from 1711 to 1597 cm^−1^ corresponded to free and bonded carbonyl stretching vibration –C=O in urethanes and uretdiones, respectively (Barikani et al. 2010; Oushabi et al. 2017; Xu et al. 2007). The absorption band that appeared at 1218 cm^−1^ could be due to C–N stretching vibrations (Hejna et al. 2017; Oushabi et al. 2017). The bands at 1067–1014 cm^−1^ were due to C–O of ether bonds of used polyols. The existence of these bands indicates the formation of –NHCOO group, which provides evidence of the success of polyurethane foam preparation (Barikani et al. 2010). In addition, there is no characteristic peak in the range of 2275–2250 cm^−1^ corresponding to N=C=O stretching, which indicates that all the isocyanate content has been consumed during foam formation (El-Molla et al. 2012). 

In order to explore the prepared materials’ optical properties, the DRS analysis of the prepared materials was conducted (Tayyab et al. 2022). The Kubelka–Munk (K–M) function (Kubelka and Munk 1931) was applied to convert the obtained reflectance spectra to absorption spectra. The optical band gap (*E*_*g*_) of the prepared materials was evaluated by the Tauc plot method (Tauc 1968) through fitting a straight line to the linear region of the spectrum and extrapolating to the energy axis. Figure 2c and d display the K–M conversion of the UV–vis DRS and Tauc plots for the prepared materials, respectively. Figure 2c shows that all the prepared materials have absorption edges in the visible region, specifically, 440 nm, 450 nm, and 460 nm for PUF, UGCN, and PUF/UGCN, respectively. This observation indicates the ability of the prepared materials to absorb visible light. In addition, it can be seen that relative to both PUF and UGCN, the absorption edge of PUF/UGCN is red shifted to 460 nm which indicates increasing its ability to absorb light over a wider range in the visible region. Finally, Fig. 2c reveals that the absorption intensity of the PUF/UGCN is upshifted implying its higher light absorption ability compared to UGCN and PUF. The band gaps calculated from the Tauc plot (Fig. 2d) are 2.91, 2.81, and 2.78 for PUF, UGCN, and UGCN/PUF, respectively. The calculated value of band gap of UGCN agrees with the literature. It has been reported that GCN has a band gap in the range 2–3 eV (Li et al. 2018). Thus, it is obvious that combining PUF and UGCN extends the visible light absorption ability of the PUF/UGCN composite and decreases the energy needed to excite electrons from the valence band to the conduction band.

**Fig. 3:**
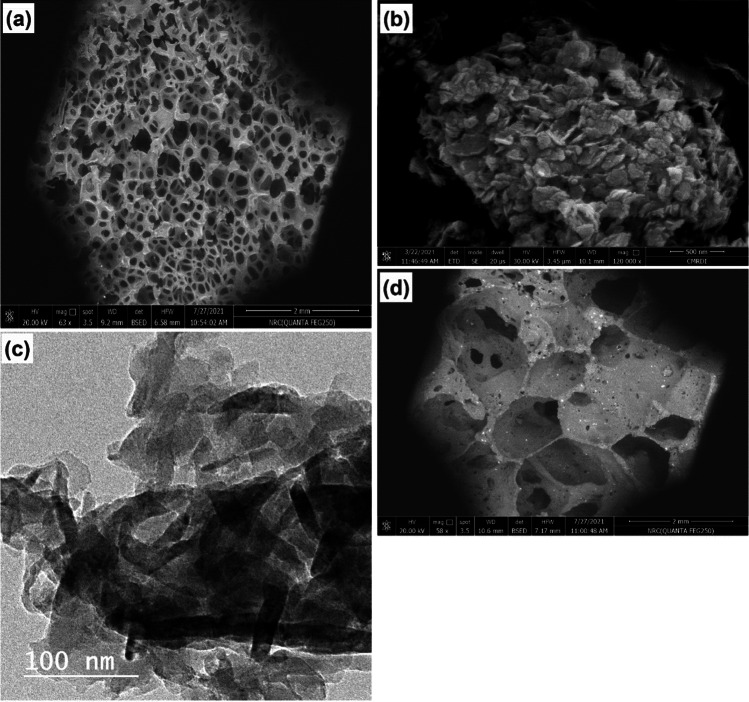
**a**, **b**, and **d** SEM micrographs of pure PUF, UGCN, and PUF/UGCN composite, respectively, and **c** TEM of UGCN

To shed more light on the prepared materials’ morphological structure, the prepared materials were visualized using SEM and TEM (Danish et al. 2021) and the micrographs are displayed in Fig. 3. From Fig. 3a, it can be observed that the pure PUF has a highly porous 3D-hierarchical structure made up of open pore channel similar to a polyhedral model and inter-connecting framework. This morphology is similar to that previously reported in the literature (Farukh and Dhawan 2016; Gavgani et al. 2016; Yuan et al. 2021). Figure 3b shows that the as-prepared UGCN exists as large aggregates composed of several ultrathin layered two-dimension sheets with a thickness of few nanometers and irregular slightly curled edges. The TEM image (Fig. 3c) further affirmed the ultrathin nanosheet morphology of the as prepared UGCN and illustrated its curled edges. Similar morphologies for UGCN have been reported before (Wang et al. 2020; Yang et al. 2017; Yu et al. 2017a). Finally, the SEM image of PUF/UGCN composite (Fig. 3d) revealed that the addition of UGCN to the PUF caused a significant decrease of the porous structure of PUF. In other words, the porosity of PUF decreased significantly upon the addition of UGCN. Figure 3d shows also the presence of numerous tinny white particles on the surface of PUF-GNC composite which might be UGCN particles.

### Photocatalytic performance of the prepared materials

**Fig. 4:**
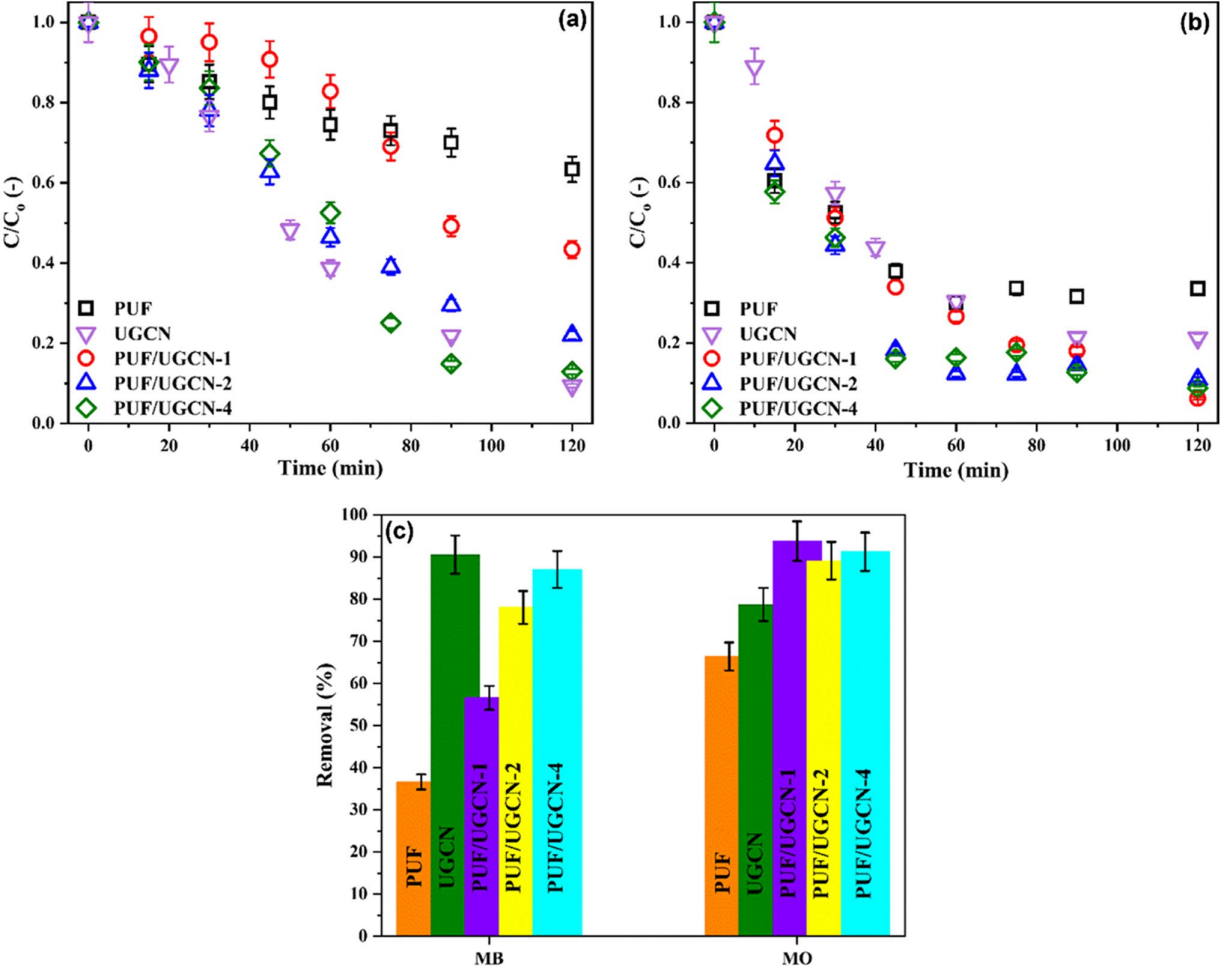
Simultaneous removal of **a** MB and **b** MO by the different prepared PUF/UGCN composite photocatalyst as a function of irradiation time and **c** after 2 h of irradiation. C_i_ 7.5 mg/L of each dye, pH_i_ 7, and dosage 1.00 g/L

The photocatalytic performance of the prepared materials was evaluated for the degradation of a mixture of a cationic and anionic dyes, specifically, MB and MO. The results are displayed in Fig. 4.

First of all, the dark adsorption and photolysis experiments showed insignificant removal of both dyes (data not shown). On the other hand, Fig. 4a and c show that increasing the ratio of UGCN in the PUF/UGCN composite photocatalyst is correlated with increasing the decolorization efficiency of MB. The MB decolorization increases monotonically as the amount of UGCN increased and reaches 87% for the composite photocatalyst with PUF/UGCN ratio 4. Notably, increasing the PUF/UGCN ratio from 2 to 4 results in slight increase of the decolorization percentage. So, no further increase in the PUF/UGCN ratio was tested and the composite photocatalyst with PUF/UGCN ratio 4 was considered the optimum. Comparing the decolorization efficiency of PUF/UGCN-4 with the pure PUF and UGCN under identical experimental conditions indicates that both pure powdered UGCN and PUF/UGCN-4 achieve almost the same decolorization percentage of MB (90% and 87%, respectively), while PUF has the lowest decolorization efficiency (36%). UGCN is the main photocatalyst component, and its visible light photocatalytic activity is well recognized (Kumar et al. 2021). On the other hand, the low photocatalytic activity of PUF has been reported before by Liu et al. (2012) who found that PUF can remove 38% of Rhodamine B (RhB) in 1 h. Noteworthy that in spite both of pure powdered UGCN and PUF/UGCN-4 achieve about the same decolorization percentage, PUF/UGCN-4 is favored by its foam form which facilitates its separation after the treatment process.

For MO, Fig. 4b and c shows that changing the PUF to UGCN ratio in the composite photocatalyst has minor effects on MO decolorization. The decolorization percentage of MO was 94%, 89%, and 91% for PUF/UGCN-1, PUF/UGCN-2, and PUF/UGCN-4, respectively. Pure PUF and UGCN decolorize 66% and 79%, respectively, of MO which indicate that both of pure PUF and UGCN can satisfactorily remove MO. However, the PUF-UGCN composites are more efficient than the pure PUF and UGCN indicating that combining PUF and UGCN improved the decolorization percentage of MO. This improvement can be correlated with the higher ability of PUF/UGCN to adsorb visible light over wider range as discussed above. Overall, Fig. 4 implies that the composite photocatalyst PUF/UGCN-4 overcomes the drawbacks of both pure PUF and UGCN as it can remove both of MB and MO efficiently and simultaneously and can be easily separated after the treatment process. Thus, the composite photocatalyst PUF/UGCN-4 was selected for further studies.

**Fig. 5:**
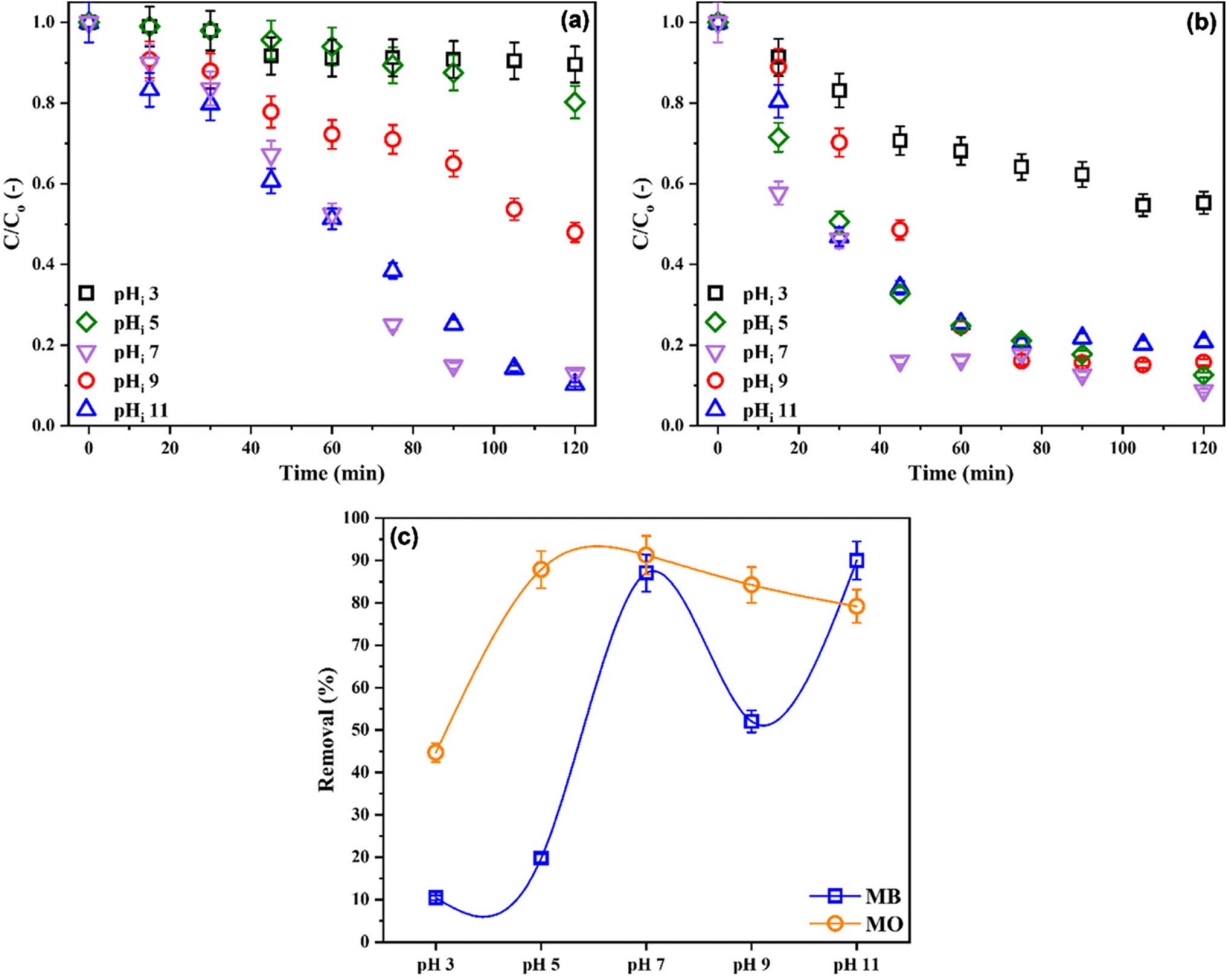
Simultaneous removal of **a** MB and **b** MO by PUF/UGCN-4 at different pH_i_ as a function of irradiation time and **c** after 2 h of irradiation. C_i_ 7.5 mg/L of each dye, and dosage 1.00 g/L

Heterogeneous photocatalysis is a surface mediated process. The first step of this process is the adsorption of the substrate on the photocatalyst surface where the light-driven chemical reaction takes place then the diffusion of the reaction by-products (Radwan et al. 2018). So, the solution pH is a critical factor that affects the rate of photocatalytic degradation since it controls the interaction between the photocatalyst and pollutant. The effects of solution initial pH on the percentage of dyes decolorized were tested using PUF/UGCN-4 as the photocatalyst and the results are displayed in Fig. 5.

The solution initial pH has remarkable effect on the decolorization percentage of MB. Increasing the initial pH from 3 to 7 causes a significant increase in MB decolorization percentage from 10 to 87%. At pH_i_ 9, the decolorization percentage decreased to 52% then recovered to 90% at pH_i_11. MB is a cationic dye with a pKa of 3.8 (Sousa et al. 2019), so at pH > 3.8, it bears positive charge in solution. At acidic pH_i_ (pH_i_ 3), PUF/UGCN-4 becomes positively charged and repels MB which hinders approaching of MB to the photocatalyst surface and results in decreasing the decolorization percentage. As the acidity of the solution decreased the positive charge on PUF/UGCN-4 surface decreases and the decolorization increases. In addition, the rate of hydroxyl radical formation, and consequently the rate of photodegradation, increases in alkaline medium due to the formation of ^−^OH ions (Kaur et al. 2013). Similar effects of pH on the degradation of MB by TiO_2_ have been reported before (Tichapondwa et al. 2020). They explained the lower removal at pH 9 by the agglomeration of TiO_2_ which results in decreasing the active surface area available for reaction and subsequent decreasing of the quantum yield.

Similar to MB, increasing the solution pH_i_ from 3 to 7 enhanced the decolorization of MO, the decolorization percentage increased from 44 to 91%. Further increase in the pH_i_ has slight negative effects on MO decolorization percentage. MO has a pKa of 3.8 (Nasrollahzadeh et al. 2018). So, at pH_i_ 3, the MO becomes positively charged and competes with hydronium ions for approaching the photocatalyst surface; thence, the photocatalytic activity decreases. Above pH 3.8, MO becomes negatively charged. Meanwhile, under acidic conditions, the surface of PUF/UGCN-4 becomes positively charged, which results in approaching of MO to the photocatalyst surface and consequently enhancing the removal of MO. Under alkaline conditions, the repulsion between negatively charged MO and PUF/UGCN-4 surface causes a reduction in the removal of MO. Similar behavior was observed by Nguyen et al., during the photocatalytic degradation of MO by ZnO/graphene oxide nanocomposite photocatalyst (Nguyen et al. 2018).

**Fig. 6:**
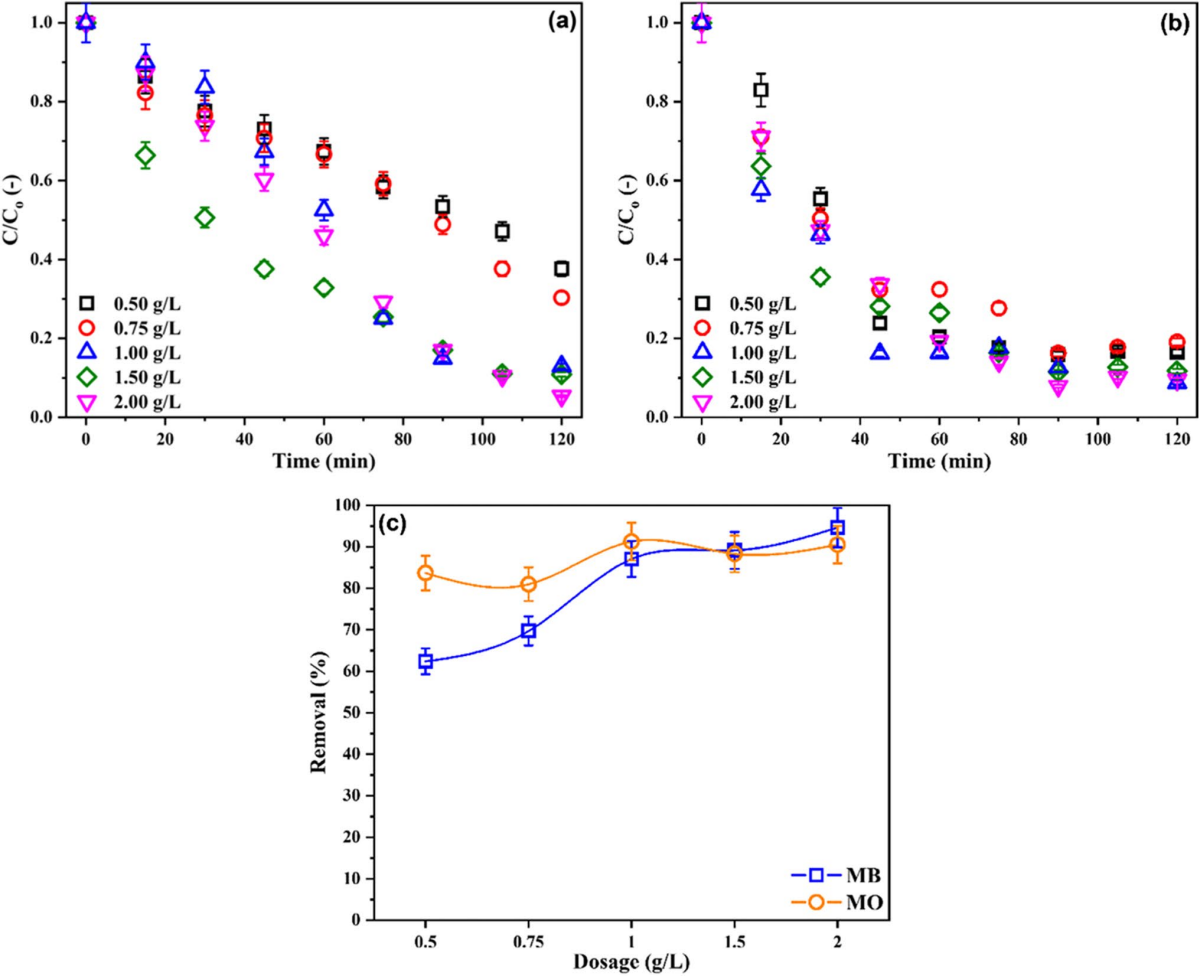
Simultaneous removal of **a** MB and **b** MO by PUF/UGCN-4 using different amounts of the composite photocatalyst as a function of irradiation time and **c** after 2 h of irradiation. C_i_ 7.5 mg/L of each dye, and pH_i_ 7

Based on these results, solution pH_i_ 7 was selected as the optimum. Therefore, the effects of the amount of the photocatalyst on the dye decolorization percentage were investigated at pH_i_ 7. The relation between the amount of photocatalyst and dye mixture decolorization percentage is shown in Fig. 6.

The effect of photocatalyst amount is more pronounced for MB than MO. In other words, the decolorization percentage of MB changed significantly as a function of photocatalyst amount, while the change in case of MO was insignificant. Particularly, increasing the photocatalyst amount from 0.50 to 1.00 g/L brought about an increase in MB decolorization percentage from 62 to 87%. Beyond a dosage of 1.00 g/L, a slight improvement in the decolorization percentage was achieved, the decolorization percentage was 89% and 94% for dosages 1.50 g/L and 2.00 g/L, respectively. In case of MO, increasing the photocatalyst dosage from 0.50 to 2.00 g/L resulted in a slight increase in the decolorization percentage from 84 to 90%. Typically, increasing the photocatalyst amount ensures more surface-active sites; consequently, more reactive oxidizing species are produced and the decolorization percentage increase. For MO, since the low amount of photocatalyst achieves high decolorization percentage, increasing the amount of photocatalyst has minor effect on the decolorization percentage.

After optimizing the preparation and experimental conditions, the decolorization efficiency of PUF/UGCN-4 towards MB and MO dyes was compared to literature. Table 1 lists some of the previously reported efficiencies of photocatalysts toward MB and MO decolorization.

**Table 1:** Comparison of methylene blue and methyl orange dye decolorization by different photocatalysts

Photocatalyst	Light source	Removal percentage (%)	Dye initial concentration (mg/L)	Photocatalyst mass (g/L)	Irradiation time (minutes)	Reference
MB						
GCN calcined at 550 °C	200 W Xenon lamp	100	10	0.5	60	Paul et al. (2019)
GCN-Ag (l mmol/10 g urea) composite	200 W tungsten lamp	96	10	0.1	120	Paul et al. (2020)
GCN-TiO_2_ (80 wt% TiO_2_)	200 W Xenon lamp	~92	20	0.7	160	Fu et al. (2014)
ZnO/rGO	500 W mercury lamp	88	5	1.5	250	Lv et al. (2011)
GCN/TiO_2_ (50 wt%) films	50 W Halogen lamp	86	10	NS	180	Boonprakob et al. (2014)
MO						
Polyetherimide/GCN floating photocatalyst	11 W table lamp	80	4	2	4080	Guo et al. (2017)
Protonated GCN/molybdenum sulphide	300 W Xenon lamp	85	20	0.4	100	Pan et al. (2021)
Thin-layer boron-doped GCN nano-sheets	Xenon lamp	94	100	2	60	Zhong et al. (2020)
Nano CdS/GCN	100 W tungsten lamp	>85	2	0.5	90	Pourshirband and Nezamzadeh-Ejhieh (2021)
PUF/UGCN-4	200 W Xenon lamp	94.6 for MB	7.5 each dye	2	120	This work
		90.5 for MO				

*NS* not stated

Table 1 clearly indicates that PUF/UGCN-4 has comparable and better decolorization of MB and MO relative to other reported photocatalysts. For instance, 1.5 g/L of the ZnO/rGO composite photocatalyst prepared by Lv et al. (2011) achieves 88% decolorization of 5 mg/L MB solution in 250 min. Similarly, only 80% of 2 mg/L MO solution was decolorized after 68 h using 2 g/L of polyetherimide/GCN floating photocatalyst developed by Guo et al. (2017). Noteworthy, the easily separable form and the floating nature of the PUF/UGCN-4 photocatalyst make it more appealing for practical application than powdered photocatalysts reported in Table 1 such as GCN (Paul et al. 2019), GCN-Ag (Paul et al. 2020), GCN-TiO_2_ (Fu et al. 2014), protonated GCN/molybdenum sulphide (Pan et al. 2021), thin-layer boron-doped GCN nano-sheets (Zhong et al. 2020), and nano-CdS/GCN (Pourshirband and Nezamzadeh-Ejhieh 2021).

**Fig. 7:**
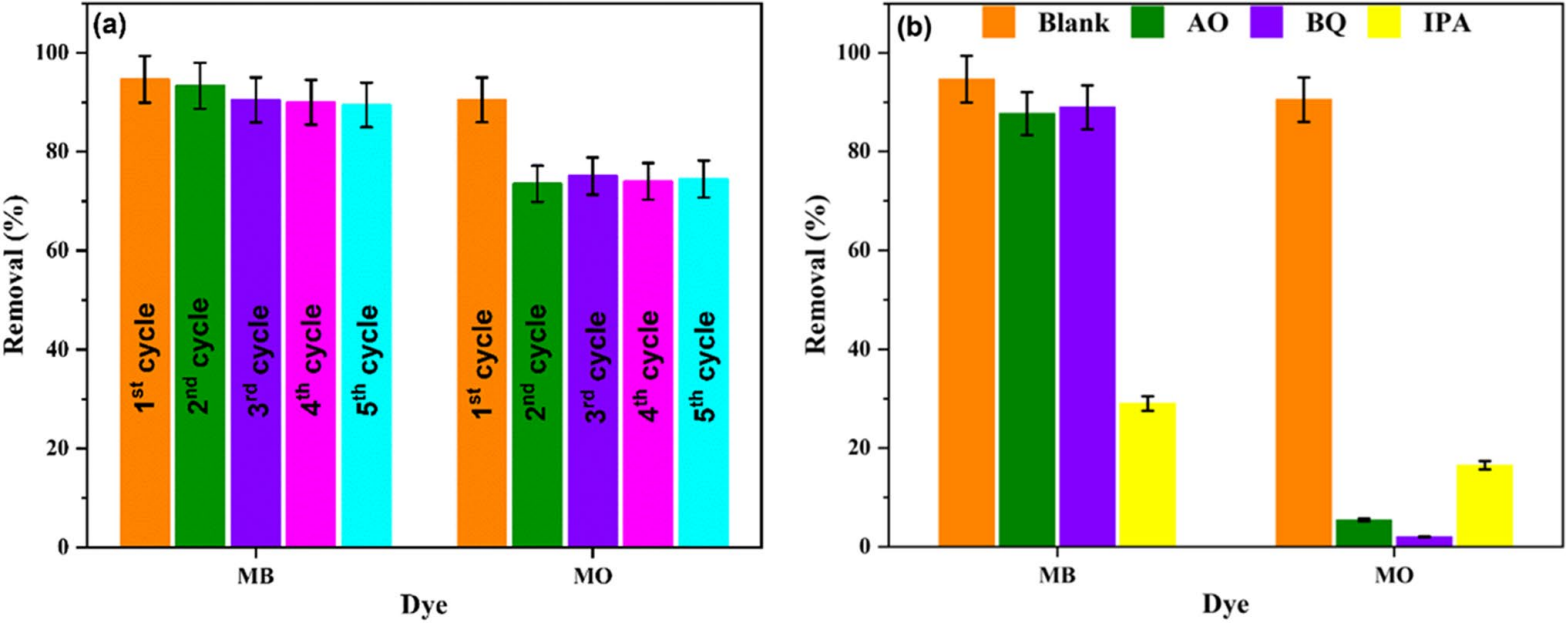
**a** Reusability of PUF/UGCN-4. **b** The effect of scavengers on the photocatalytic degradation of dyes mixture using PUF/UGCN-4. C_i_ 7.5 mg/L of each dye, pH_i_ 7.0, dosage 2.00 g/L, and irradiation time 2 h

The reusability of a photocatalyst is a vital character that determines its economic visibility and practical applicability. Therefore, the regeneration and reuse of PUF/UGCN-4 were tested at pH_i_ 7 using 2.00 g/L and 7.5 mg/L of each dye in a mixture solution. After irradiation for 2 h, the photocatalyst was separated and washed with ethanol solution (1:1), DW, 0.1N HCl, DW, 0.1N NaOH then DW. After that, the photocatalyst was dried, and the photocatalytic degradation process was performed again under the same first conditions. Figure 7a shows that after five runs, a minor reduction in the decolorization percentage of MB was observed. Specifically, the decolorization percentage decreased from 94 to 89.5% after five reusing cycles. This result manifests the excellent photocatalytic performance durability under irradiation and degradation process of MB. On the other hand, a notable reduction in the decolorization percentage of MO was observed in the second run, decolorization percentage decreased from 90 to 73%, then remained almost constant (74.5%) thereafter, indicating that the photocatalytic degradation activity of the PUF/UGCN-4 toward MO was retarded after the first use then remained constant.

**Fig. 8:**
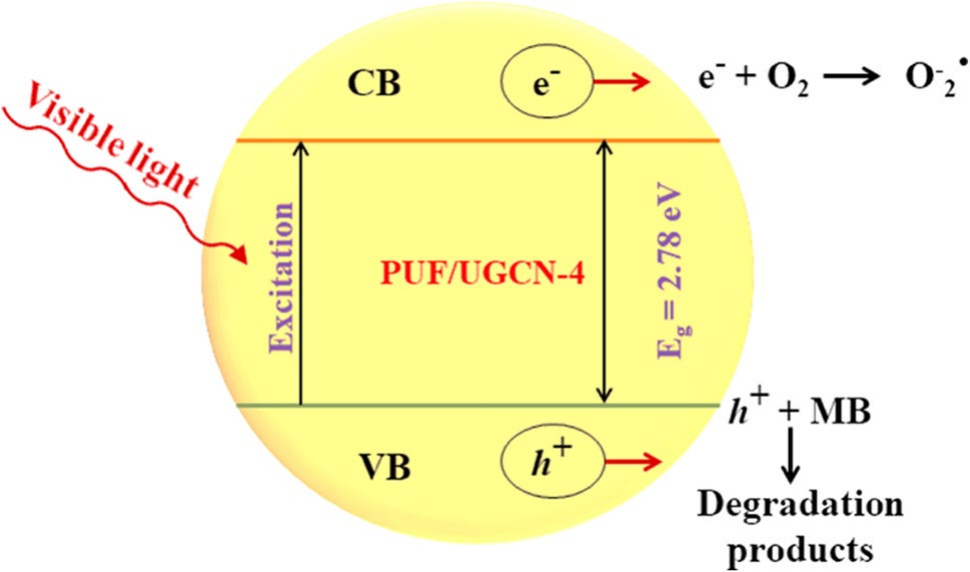
Proposed mechanism of reactive species generation and dye degradation by PUF/UGCN-4 photocatalyst

In order to understand the photocatalytic reaction mechanism, radical trapping experiments were performed and the decolorization percentage in the presence of scavengers was evaluated (Chankhanittha and Nanan 2021). Ammonium oxalate (AO), benzoquinone (BQ), and isopropanol (IPA) were used as hole, superoxide radical, and hydroxyl radical scavengers, respectively (Zheng et al. 2019). Figure 7b displays the obtained results. It can be seen that the addition of AO and BQ have minor effect on the decolorization percentage of MB, while the addition of IPA reduced the decolorization percentage significantly. These results indicate that hydroxyl radicals play the key role in the degradation of MB. On the other hand, the addition of AO, BQ, and IPA decreases the decolorization percentage significantly. Therefore, holes, superoxide radicals, and hydroxyl radicals are essential in the photocatalytic degradation of MO. The superoxide radicals have greater influence than holes which in turn more influent than hydroxyl radicals. Therefore, the photocatalytic degradation of dyes mixture can be presented as schematized in Fig. 8 and described by the following equations.

1 $$\mathrm{PUF}/\mathrm{UGCN}\ \overset{\mathrm{Visible}\ \mathrm{light}}{\to }\ {e}^{-}+{h}^{+}$$

2 $${e}^{-}+{\mathrm{O}}_2\ \left(\mathrm{dissolved}\right)\to {}^{\bullet }{\mathrm{O}}_2^{-}$$

3 $${}^{\bullet }{\mathrm{O}}_2^{-}+{\mathrm{H}}_2\mathrm{O}\to {}^{\bullet}\mathrm{OH}+{}^{-}\mathrm{OH}+1/2\ {\mathrm{O}}_2$$

4 $${}^{\bullet}\mathrm{OH}+\mathrm{MB}\to \mathrm{Degradation}\ \mathrm{products}$$

5 $${}^{\bullet }{\mathrm{O}}_2^{-}+\mathrm{MO}\to \mathrm{Degradation}\ \mathrm{products}$$

6 $${h}^{+}+\mathrm{MO}\to \mathrm{Degradation}\ \mathrm{products}$$

7 $${}^{\bullet}\mathrm{OH}+\mathrm{MO}\to \mathrm{Degradation}\ \mathrm{products}$$

The band gap of PUF/UGCN-4 enables it from absorbing visible light radiation as discussed above. Therefore, irradiating the PUF/UGCN-4 with visible light initiates the photocatalytic reaction by the generation of *e*^*−*^ and *h*^*+*^ (Eq. 1). The CB of UGCN is about −1.04 V (Thanh Tung et al. 2022) which enables the *e*^−^ in the CB of UGCN to react with oxygen to produce superoxide radicals (Eq. 2). These superoxide radicals react with water to produce hydroxyl radicals (Eq. 3). The generated hydroxyl radicals degrade MB, while hydroxyl radicals, holes, and superoxide radicals contribute in the degradation of MO to different extents (Eqs. 4–7).

## Conclusions

Developing new easily separable materials that can efficiently decontaminate water is highly demanded. In this study, we targeted the preparation of a visible light active photocatalyst that can efficiently and simultaneously remove anionic and cationic dyes from contaminated water and can be easily separable after the treatment process. Our strategy was to immobilize a metal-free visible light active photocatalyst, namely, ultrathin graphite carbon nitride nanosheets, onto a low-cost three-dimensional porous flexible support, namely, polyurethane foam. Therefore, polyurethane foam, ultrathin graphitic carbon nitride, and a series of their composite were prepared, fully characterized, and their photocatalytic activity for the decolorization of a mixture of anionic and cationic dyes was investigated. The transmission electron microscopy micrograph demonstrated the ultrathin nanosheet structure of the as-prepared graphitic carbon nitride, and the scanning electron microscope micrograph showed its agglomeration into large clusters. The composite photocatalyst combined the X-ray diffraction, and Fourier transform infrared features of the individual materials. The diffuse reflectance spectroscopy analysis illustrated that the composite material has better visible light absorption than the individual materials. As a photocatalyst, the composite material outperformed the pure components in several aspects. First, compared to powdered ultrathin graphitic carbon nitride nanosheets, it kept the same photocatalytic activity toward methylene blue and had better activity toward methyl orange. Second, it was reusable for five cycles which makes it economically and practically visible. Third, it is easily separable because of it foam form. Future work should focus on the development of new eco-friendly, low-cost, easily separable, and highly efficient photocatalysts for water decontamination.

## Data Availability

Note applicable.
